# CT features of feline cystic bronchiectasis forming mass lesions

**DOI:** 10.1177/20551169231217866

**Published:** 2024-01-19

**Authors:** William J Moorhead, Wilfried Mai, Jennifer A Reetz, Silke Hecht, Peter G Noel

**Affiliations:** 1 Department of Clinical Sciences and Advanced Medicine, School of Veterinary Medicine, University of Pennsylvania, Philadelphia, PA, USA; 2 Department of Small Animal Clinical Sciences, College of Veterinary Medicine, University of Tennessee, Knoxville, TN, USA; 3 Parallax Teleradiology, Hastings-on-Hudson, NY, USA

**Keywords:** Bronchiectasis, pulmonary mass, CT, bronchial

## Abstract

**Case series summary:**

Cystic bronchiectasis was diagnosed in three cats with known histories of chronic coughing using CT and histopathology. CT of the lungs revealed large space-occupying lesions that compressed and displaced unaffected pulmonary parenchyma and vessels. The masses were soft tissue attenuating in two cases and gas-cavitated with areas of dependent fluid in one case. All three cats were found to have mineral attenuating material in lesions and in other dilatated airways. Generalized bronchial wall thickening was also present and indicative of chronic lower airway disease. These findings were supported by histopathology showing inflammatory changes and dilatated airways in the collected tissues. In the two cases in which post-contrast CT series were acquired, the lesions had rim-enhancement.

**Relevance and novel information:**

Cystic bronchiectasis is a rare presentation of bronchiectasis in cats and may mimic a pulmonary mass lesion, which could be mistaken for neoplasia or abscessation. The lack of central enhancement or presence of gas cavitation on CT, concurrent presence of diffuse bronchial wall thickening, other areas of bronchiectasis and the presence of broncholithiasis may alert the clinician to the possibility of cystic bronchiectasis related to chronic lower airway disease.

## Introduction

Acquired bronchiectasis is defined as irreversible, pathologic dilatation of the airways resulting from destruction of the structural components of the bronchial walls due to chronic inflammation.^1,2^ In human medicine, three types of bronchiectasis are described: cylindrical (also called tubular), varicose and cystic (also called saccular).^1–4^ Cylindrical bronchiectasis is defined as smooth dilatation of the bronchi, varicose bronchiectasis as beaded dilatation of the bronchi with irregular contours and cystic (saccular) bronchiectasis as bronchial dilatation greater than 1 cm or progressive dilatation of airways leading to grape-like clusters that can contain fluid from retained secretions.^3,4^ Cylindrical bronchiectasis is the most common form of bronchiectasis in dogs and cats, and affects more commonly the larger, thick-walled bronchi, whereas saccular bronchiectasis affects intermediate-sized bronchi.^2^ Varicose bronchiectasis has not been described in dogs and cats.^2^

Cystic bronchiectasis may be considered a severe manifestation of saccular bronchiectasis affecting terminal bronchi.^2^ This form has been reported in one dog.^1^ Although there have been reports of bronchiectasis in cats,^2^ the lesions do not fit the description of cystic bronchiectasis.

The purpose of this case series was to describe the CT features seen in feline patients with confirmed cystic bronchiectasis forming mass-like lesions on thoracic imaging.

## Case series description

The medical records from the University of Tennessee College of Veterinary Medicine, the Schwarzman Animal Medical Center of New York City and the University of Pennsylvania School of Veterinary Medicine were searched for cats with surgically and histopathologically confirmed cystic bronchiectasis that received CT before surgery.

### Case 1

An 11-year-old neutered male domestic shorthair cat presented with a chronic cough. The patient was initially treated with prednisone with transient improvement of respiratory signs. Thoracic radiographs showed a large oval-shaped soft-tissue opaque mass in the left caudal lung lobe. On the ventrodorsal view of the thorax, there were also several soft-tissue nodular and tubular opacities adjacent to the larger mass lesion in the left caudal lung lobe. CT was performed to further evaluate the lesion.

**Figure 1:**
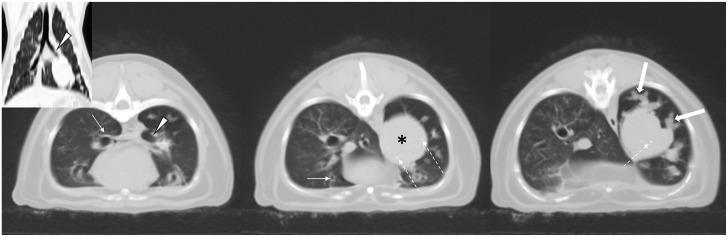
Serial transverse CT images (left to right, cranial to caudal) of the caudal thorax of the cat in case 1; the insert shows a reformatted image in the dorsal plane. Images are pre-contrast and displayed in a lung window. There is a large rounded soft-tissue attenuating mass in the left caudal lung lobe (asterisk) that appears continuous with the left caudal bronchus (arrowhead). The mass contains pinpoint mineral attenuating foci (dashed arrows). Caudal to the mass, there are tubular fluid-filled tapering structures interpreted as bronchi filled with fluid (thick arrows). There is generalized bronchial wall thickening (thin solid arrows)

CT examination (without contrast) revealed generalized bronchial wall thickening and a 3 cm diameter, smoothly marginated rounded soft-tissue attenuating (45 Hounsfield units [HU]) mass with multifocal pinpoint mineral densities in the caudal left lung lobe (Figure 1). The left caudal lobar bronchus was narrowed and on sequential images could be traced into the cranial extent of the mass lesion. Extending from the mass lesion, there were many tubular soft-tissue attenuating structures with a branching pattern consistent with dilatated fluid-filled bronchial lumens. No post-contrast imaging was obtained.

A thoracotomy and left caudal lung lobectomy were performed. Histopathology showed severe multifocal bronchiectasis of the bronchi and larger bronchioles, which were filled with eosinophilic debris with cholesterol clefts, sloughed cells and mucus. There was peribronchial gland hyperplasia with mucus distension. The peribronchial interstitium had moderate lymphoplasmacytic infiltrates with eosinophils and occasionally contained mucus accompanied by lymphoplasmacytic and histiocytic infiltrates, fibroplasia and mineral deposits. There was atelectasis and compression of alveoli throughout the sections, most markedly near the distended airways. Based on the imaging and histopathological findings, the final diagnosis was chronic lower airway disease with severe cystic (or saccular) bronchiectasis in the left caudal lung lobe. Mycoplasma, bacterial and fungal culture of surgical samples were all negative and fecal analysis did not reveal lung worm larvae. The cat recovered from surgery uneventfully and was discharged with long-term medical management for presumed asthma.

### Case 2

A 14-year-old neutered male domestic shorthair cat presented with a 2-month history of intermittent lethargy that progressed over the week before the visit. The patient was known to have feline asthma that was managed with daily fluticasone nasal spray.

**Figure 2:**
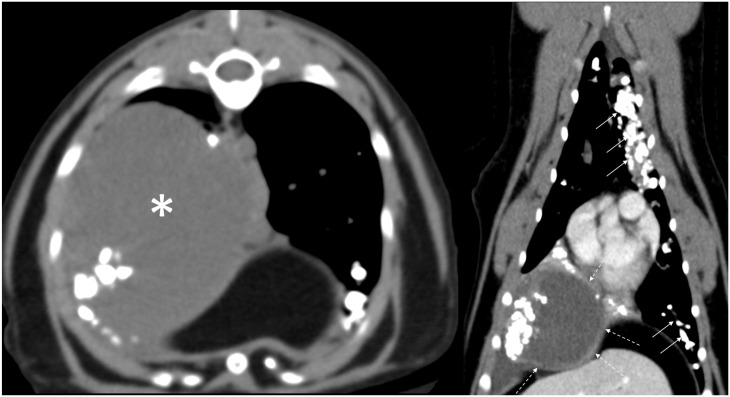
Transverse (left) and reformatted dorsal plane (right) CT images of the thorax of the cat in case 2; the image on the left is pre-contrast and image on the right is post-contrast; both are displayed in a soft-tissue window. There is a large soft-tissue attenuating mass in the right caudal lung lobe (asterisk) with rim enhancement seen on post-contrast images (dashed arrows). Numerous small mineral bodies are seen in the mass as well as arranged along numerous bronchial lumens (solid arrows), consistent with broncholithiasis

CT examination revealed a 6 cm diameter soft-tissue attenuating mass (25–30 HU) in the right caudal lung lobe, which had rim enhancement on post-contrast imaging and a non-enhancing center (Figure 2). In the right ventrolateral aspect of the non-enhancing central aspect of the mass, there were amorphous mineral densities. The right caudal lobar bronchus and artery were displaced by the mass, and the dorsomedial aspect of the right caudal lung lobe was atelectatic. The right cranial lobar bronchus was pathologically dilatated and variably filled with non-enhancing soft-tissue attenuation mixed with mineral densities. Throughout all lung lobes, there were round to linear, endobronchial mineral attenuating foci. These findings were worse in the left cranial lobe where they were surrounded by non-enhancing lobular soft-tissue attenuating regions. Two cranial mediastinal lymph nodes were mildly enlarged and there was no tracheobronchial lymphadenopathy.

A right caudal lung lobectomy was performed, with uneventful recovery. On gross examination, purulent material drained from the excised lung lobe. Histopathologically, there was severe bronchiectasis with parenchymal rupture with mineral concretions and regions of chronic necrotizing and suppurative pneumonia. No culture or long-term follow-up was available.

### Case 3

An 11-year-old neutered male domestic shorthair cat presented with a 1-year history of cough, progressing from exercise-induced to coughing at rest. Wheezes were heard on thoracic auscultation. Thoracic radiographs showed a diffuse bronchointerstitial lung pattern with multifocal bronchiectasis and multifocal mineralization in the caudodorsal lung field interpreted as broncholithiasis and/or dystrophic mineralization of lung parenchyma. There was also a large cavitated mass with a thick, irregular rim in the left caudal lung lobe.

**Figure 3:**
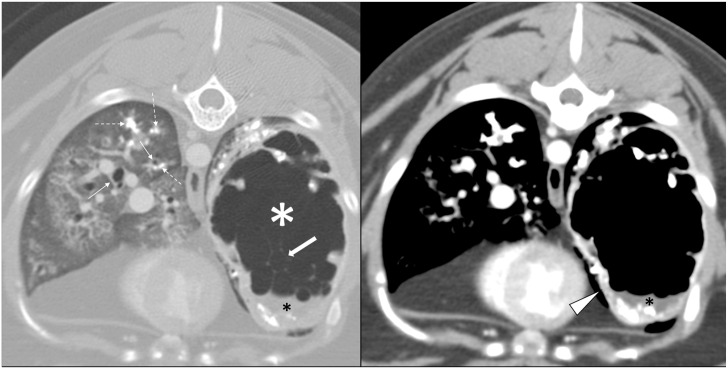
Transverse CT images of the caudal thorax of the cat in case 3; the image on the left is a high-resolution image in a lung window while on the right is a post-contrast image in a soft-tissue window. A large gas-cavitated mass is present in the left caudal lung lobe (white asterisk); thin internal septations are present in the mass (thick arrow) and soft-tissue attenuating non-enhancing material (black asterisk) is seen in the dependent portion of the mass consistent with fluid. Thin rim enhancement is noted after contrast administration (arrowhead). Numerous mineral foci are present in the lumen of small airways (dashed arrows) consistent with broncholithiasis. There is diffuse bronchial wall thickening (thin solid arrows)

A CT examination revealed a large, cavitated, gas-filled and rim-enhancing mass lesion in the left caudal lung lobe, which appeared to be continuous with a lobar bronchus (Figure 3). Within the medial, ventral and cranial aspect of the lesion, there were internal septations. The lesion had a thick, irregular rim with multifocal areas of mineralization either within the rim or closely associated with the rim in the lung parenchyma around the lesion. There was fluid-attenuating, non-contrast enhancing material in the dependent portion of the mass, consistent with fluid. There was a mass effect with a mediastinal shift to the right and flattening of the left diaphragmatic crus. Other findings included multifocal bronchial wall thickening and cylindrical bronchiectasis, with mineral and soft-tissue attenuating material present in some bronchi (broncholiths).

A left lateral thoracotomy for left caudal lung lobectomy was performed. At surgery, a large, cavitated air-filled lesion occupying the entire left caudal lung lobe was noted. Histopathology showed changes consistent with feline asthma, including bronchiectasis, chronic bronchitis, alveolar dysplasia, atelectasis, peripheral emphysema and mucosal gland hyperplasia, with no evidence of neoplasia. Bacterial and mycobacterial cultures were negative, while *Penicillium* species was grown on fungal culture, but this was considered a contaminant. The cat recovered well after surgery. No long-term follow-up was available.

## Discussion

In this study, we describe the imaging appearance of cystic bronchiectasis, which appeared as soft-tissue attenuating or gas-cavitated large mass lesions. The imaging features had differential diagnoses of neoplasia, abscesses or granulomatous lesions. This report brings attention to the fact that large pulmonary mass lesions can represent atypical presentation of bronchiectasis, carrying a better prognosis than pulmonary neoplasia.

Although the imaging features can be misleading, the concurrent presence of generalized bronchial wall thickening, bronchiectasis and broncholithiasis should raise the possibility that the mass lesion may represent cystic bronchiectasis. Careful tracing of the bronchi in the vicinity of the mass lesion may also be helpful; as we saw in two cases, a bronchus seemingly merged with the mass lesion when evaluating sequential images. For solid-looking lesions, the internal attenuation was higher than that of pure fluid, which is consistent with mucoid content mixed with cellular debris, as was observed histopathologically.

In veterinary medicine, multiple terms have been used to describe bronchiectasis, including cylindrical, tubular, saccular, varicose and cystic.^1–6^ Cylindrical/tubular bronchiectasis has been reported to be the most common type in dogs.^1,3,5^ Cystic bronchiectasis has been described in one dog,^1^ and though this was a radiographic description, the general appearance of the lesion was similar to the cats reported herein, particularly case 3. Using the terminology in human medicine and comparing the lesions seen in these cats to that in humans^3^ and in the previously reported dog,^1^ the lesions in these cats were also considered a form of cystic bronchiectasis. Other areas of bronchiectasis seen in case 3 were considered to be cylindrical, as they were smoothly dilatated and non-tapering.

Damage to the epithelium and cilia of the bronchi, caused by foreign material, infection or inflammation, can lead to the accumulation of fluid and secretions, further predisposing patients to secondary infection and inflammation.^6^ With chronic inflammation, the bronchial walls are constantly under stress and repeatedly damaged, resulting in the weakening of bronchi and subsequent dilatation by normal respiratory expansion of the thorax.^1,6^ The degree of expansion likely correlates with the amount of cartilage in the wall.^1^ This may result in more severe, progressive dilatation in terminal bronchi, leading to the cystic changes as seen in these three cases.

All three cats in this report were found to have mineral-attenuating foci in the dilatated airways, which is reported to be a common finding secondary to feline chronic inflammatory lower airway disease, which also includes bronchiectasis.^7^ All three cats were older and were known to have a 1–2-year history of chronic cough; therefore, the presence of these mineral opacities is likely the result of a chronic process resulting in the formation of mineral concretions in the airways, known as broncholithiasis.

## Conclusions

Although cylindrical (tubular) bronchiectasis is the most common form in cats, cystic bronchiectasis is a rare manifestation of this condition, which could present on radiographic or CT imaging studies as a mass lesion and could therefore be mistaken for neoplasia or primary pulmonary abscessation. The concurrent presence of diffuse bronchial wall thickening, other areas of bronchiectasis and broncholithiasis should alert the clinician to the possibility that the mass lesion may be non-neoplastic and associated with chronic lower airway disease. CT is an effective tool in the diagnosis of feline cystic bronchiectasis as it allows for a more precise assessment of the relationship of the mass lesion to the bronchial tree, as well as a better characterization of concurrent airway changes (bronchial wall thickening, other areas of bronchiectasis and broncholithiasis), which may be helpful in prioritizing the differential diagnosis.
